# Staged retroauricular flap for helical reconstruction after Mohs micrographic surgery*

**DOI:** 10.1590/abd1806-4841.20164733

**Published:** 2016

**Authors:** Felipe Bochnia Cerci

**Affiliations:** 1 Hospital Santa Casa de Curitiba – Curitiba (PR), Brazil

**Keywords:** Mohs surgery, Skin neoplasms, Ear neoplasms, Surgical flaps

## Abstract

Staged retroauricular flap is a great option for full-thickness defects along the helical rim and antihelix. Donor site consists of the posterior ear, postauricular sulcus and mastoid area. The advantages of this flap include hidden donor scar, donor tissue similarity and rich vascularity. We present a case of collision tumor on the left helix treated with Mohs micrographic surgery and the resulting full-thickness defect repaired with a staged retroauricular flap. This flap is an effective technique for full-thickness helical defect repair with relatively little operative morbidity. High esthetic and functional results may be obtained restoring the ear size and shape.

## INTRODUCTION

Staged flaps are excellent choices for reconstruction of ear defects of varying complexity and location. Wounds along the helical rim, lobule, antihelix and conchal bowl may be resurfaced with either staged pre-auricular or retroauricular flaps.^1,2^

The staged retroauricular flap (SRF) is more often used and is most applicable to repair full-thickness helical rim and antihelix defects. Donor site consists of the posterior ear, postauricular sulcus and mastoid area. Advantages of this flap include donor tissue similarity, rich vascularity and hidden donor scar.^1,2,3^ Its main disadvantage is the necessity for a two-stage procedure.

## CASE REPORT

A 52-year-old male patient was referred to the department of dermatology to evaluate an erythematous plaque (1.6 cm x 1.4 cm) involving the left helix. Biopsy revealed a collision tumor consisting of an infiltrative basal cell carcinoma and an invasive squamous cell carcinoma.

**Figure 1:**
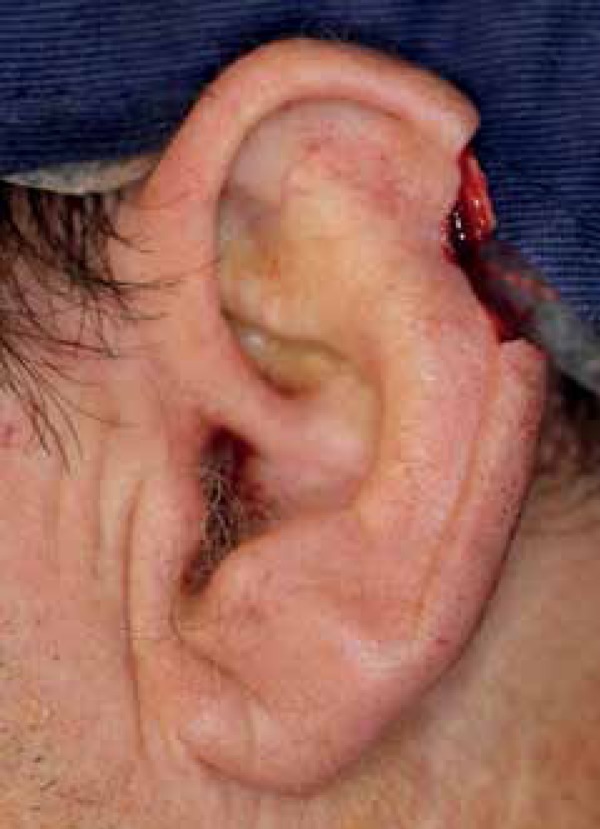
Full - thickness defect involving the left helix after Mohs surgery

The patient was submitted to Mohs micrographic surgery under local anesthesia with bupivacaine and lidocaine. After two stages, clear margins were achieved. The resulting defect measured 1.9 cm x 1.7 cm and affected the left helix (Figure 1). Part of the cartilage was removed leading to a full-thickness defect. Due to the horizontal configuration of the defect, and after discussing outcomes with the patient, it was opted for a staged retroauricular flap repair.

**Figure 2:**
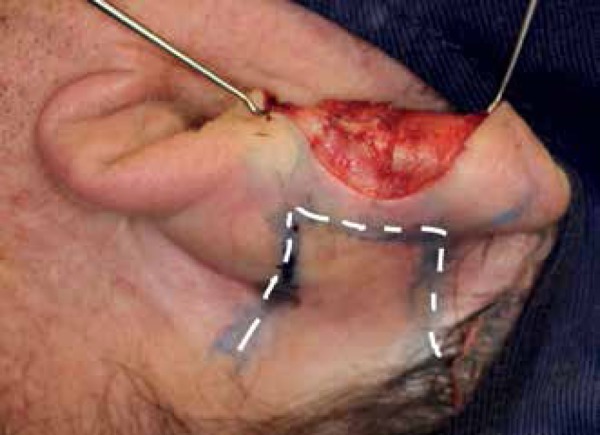
Flap design (dashed line). Donor sites include the postauricular ear, sulcus, and mastoid area. The vertical height of the flap equals that of the defect on the helical rim. The pedicle base is slightly widened for a better blood supply

**Figure 3:**
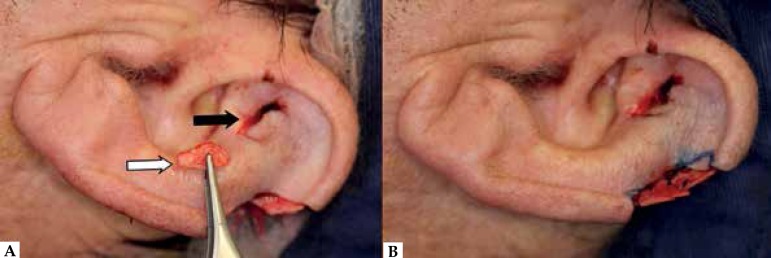
**A:** Cartilage graft (white arrow) harvested from the antihelix (black arrow). **B:** Cartilage graft sutured into place

First, assessment of the anterior and posterior components of the primary defect was made. The auricle was pressed against the mastoid to outline the vertical length of the flap (Figure 2). Then, in order to recreate the helix curvature, a cartilage graft was harvested from the ipsilateral antihelix and sutured into the defect (Figure 3).

**Figure 4:**
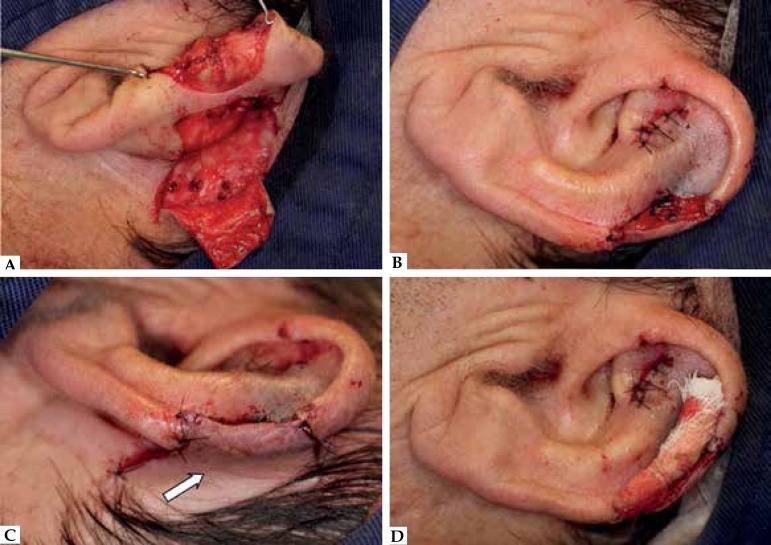
**A:** Flap elevated above perichondrium and fascia to ensure a rich blood supply. **B:** Anterior portion of the defect partially left to heal by secondary intention. **C:** Postauricular pedicle (arrow). **D:** Gauze sutured into the sulcus to help reestablish a natural- -looking proximal helical rim curvature

The remaining of the flap was designed with two horizontal lines from the retroauricular skin towards the hairline with progressive pedicle widening. The flap was elevated above the perichondrium and fascia to ensure rich blood supply (Figure 4A). The mastoid scalp was slightly undermined to increase flap mobility. The distal flap was thinned to obtain an adequate thickness match and sutured into the defect. Part of the anterior defect was left to heal by secondary intention (Figure 4B-C) and a gauze was sutured (for 3 days) at the antihelical groove to help reestablish a natural-looking proximal helical rim curvature (Figure 4D).

Donor site was left to heal by secondary intention. Gauze was also inserted into the postauricular tunnel for hemostasis, comfort and prevention of contact between primary and secondary wounds.

**Figure 5:**
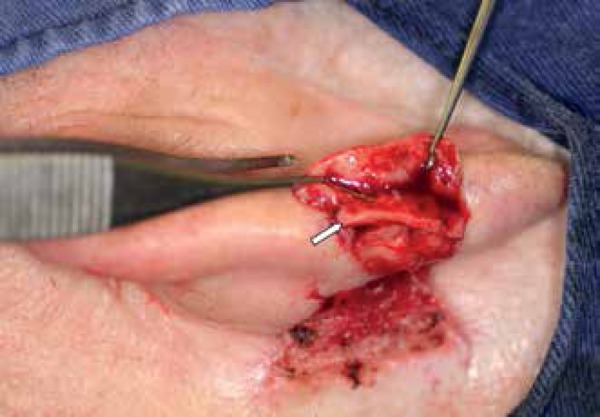
“Flap thinning during the second stage (arrow)

**Figure 6:**
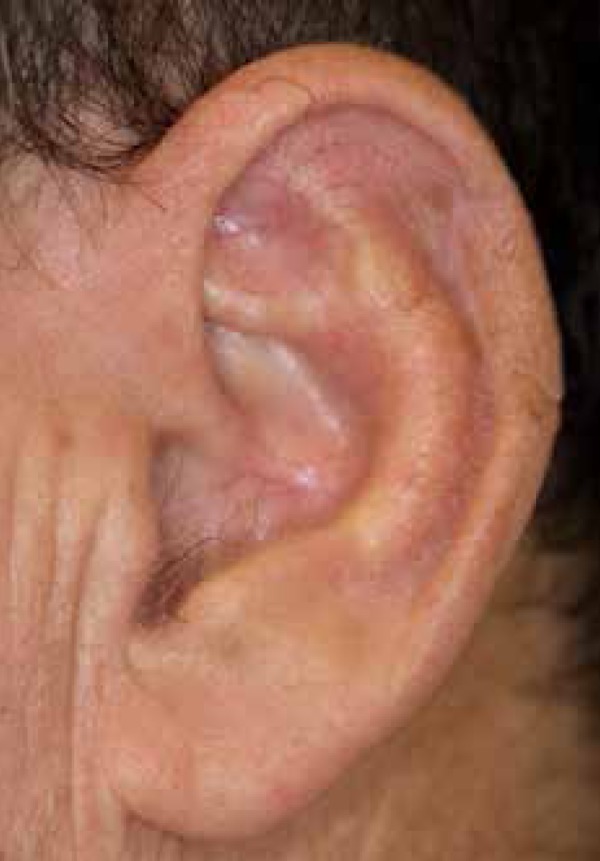
Two months postoperative. Contour, substance, and integrity of the helical rim have been maintained

After 3 weeks, the flap was divided at its posterior attachment and thinned to provide precise fit and contour. The edges were curled over the helical rim and sutured onto the posterior primary defect (Figure 5). The remaining pedicle was discarded and the donor area was left to heal by secondary intention. Two months postoperatively, the patient had a satisfactory result with helical rim restoration (Figure 6).

## DISCUSSION

Auricular reconstruction may be challenging due to the complex topography of the ear. Distortions in the ear architecture or symmetry can affect facial aesthetics.^2^ Several reconstructive options are available and should be individualized according to each case and surgeon´s preference. Skin grafts, one-stage flaps, or secondary intention healing usually offer good results on the ear.^3,4^ Ear staged flaps are often reserved for complex defects, cosmetically sensitive patients or for functional restoration.^5^

The staged retroauricular flap (SRF) is a random-pattern flap with a rich vascular supply based on branches of the posterior auricular, superficial temporal and occipital arteries. The main disadvantage of this flap is the need for two stages to complete the procedure.^1,2^ Posterior ear, postauricular sulcus and mastoid area provide good skin color/texture match for excellent cosmetic results.^5^

Although full-thickness defects may be restored in single-stage procedures, SRF can restore the ear to its previous size and shape.^6^ In the present case, a wedge resection was also considered. However, due to the horizontal orientation of the defect, a significant non-affected area would have been removed, leading to a substantial reduction in the ear size.

Loss of cartilage can be tolerated without adverse sequelae for most ear defects, considering that the ear is an excellent donor site for cartilage grafts in nasal reconstruction.^7,8^ If the primary defect involves complete loss of a portion of the helical rim and a small amount of cartilage, the flap may be folded onto itself to provide adequate thickness and support without a cartilage graft. For larger defects, cartilage replacement may be required for support. In these cases, cartilage may restore esthetic contour and/or structural integrity and stability.^5^

Recreation of the helical rim curvature is challenging. Distinct techniques may be employed, including basting sutures, jelly roll flap and secondary intention healing. Basting sutures are more successful during the first stage to recreate the helical sulcus since wound healing and flap contraction at the second stage may distort the sulcus.^2^ The jelly roll technique consists of doubling up the skin flap into a roll to simulate the soft tissue that overlies the cartilage. Horizontal mattress sutures behind the flap leading edge place the helical skin under compression and create redundancy.^9^ Secondary intention healing of the anterior portion of the defect (if limited in size) naturally helps to recreate the sulcus, as in the present case. Regardless of the technique implemented, flaps should not be placed under tension since this will attenuate and flatten the sulcus.

The donor area is left to heal entirely by secondary intention or may be partially covered with the remaining pedicle after the second stage.^1,2,6^ Safety of performing SRF under local anesthesia has been well documented with low complication rates when applied with appropriate techniques.^10^ SRF is an effective repair method for full-thickness helical defects that has relatively little operative morbidity. High esthetic and functional results may be obtained restoring the ear size and shape.
